# RIG-I Deficiency Promotes Obesity-Induced Insulin Resistance

**DOI:** 10.3390/ph14111178

**Published:** 2021-11-17

**Authors:** Gabsik Yang, Hye Eun Lee, Jin Kyung Seok, Han Chang Kang, Yong-Yeon Cho, Hye Suk Lee, Joo Young Lee

**Affiliations:** 1 College of Pharmacy, The Catholic University of Korea, Bucheon 14662, Korea; yanggs@woosuk.ac.kr (G.Y.); esthel0513@catholic.ac.kr (H.E.L.); jkseok@catholic.ac.kr (J.K.S.); hckang@catholic.ac.kr (H.C.K.); yongyeon@catholic.ac.kr (Y.-Y.C.); sianalee@catholic.ac.kr (H.S.L.); 2 Department of Pharmacology, College of Korean Medicine, Woosuk University, Jeonju 55338, Korea; 3 BK21FOUR Team, College of Pharmacy, The Catholic University of Korea, Bucheon 14662, Korea

**Keywords:** pattern-recognition receptors, obesity, metabolic syndrome, metainflammation, ER stress

## Abstract

Inflammation and immunity are linked to the onset and development of obesity and metabolic disorders. Pattern recognition receptors (PRRs) are key regulators of inflammation and immunity in response to infection and stress, and they have critical roles in metainflammation. In this study, we investigated whether RIG-I (retinoic acid-inducible gene I)-like receptors were involved in the regulation of obesity-induced metabolic stress in RIG-I knockout (KO) mice fed a high-fat diet (HFD). RIG-I KO mice fed an HFD for 12 weeks showed greater body weight gain, higher fat composition, lower lean body mass, and higher epididymal white adipose tissue (eWAT) weight than WT mice fed HFD. In contrast, body weight gain, fat, and lean mass compositions, and eWAT weight of MDA5 (melanoma differentiation-associated protein 5) KO mice fed HFD were similar to those of WT mice fed a normal diet. RIG-I KO mice fed HFD exhibited more severely impaired glucose tolerance and higher HOMA-IR values than WT mice fed HFD. IFN-β expression induced by ER stress inducers, tunicamycin and thapsigargin, was abolished in RIG-I-deficient hepatocytes and macrophages, showing that RIG-I is required for ER stress-induced IFN-β expression. Our results show that RIG-I deficiency promotes obesity and insulin resistance induced by a high-fat diet, presenting a novel role of RIG-I in the development of obesity and metabolic disorders.

## 1. Introduction

Obesity is associated with chronic inflammation, which contributes to other diseases, such as atherosclerosis, type 2 diabetes, and fatty liver diseases. It is now well established that metabolism and immunity are closely linked and that metabolic stress leads to immune imbalance, as there are many factors mediating both metabolism and immunity. Dysregulation of metabolism and immunity is interconnected, as inflammatory signaling pathways are activated by metabolic stresses. ER stress and mitochondrial production of ROS are increased by dysregulation of metabolism, leading to the activation of inflammatory signaling pathways. Therefore, understanding the mechanism by which obesity and immunity are linked would provide important clues for the design of new preventive or therapeutic strategies for chronic inflammatory and metabolic disorders.

Pattern-recognition receptors (PRRs) play critical roles in regulating the immune system and inflammatory responses by recognizing pathogen-associated molecular patterns (PAMPs) and danger-associated molecular patterns (DAMPs). Persistent inflammation in tissues by DAMPs is accompanied by the secretion of cytokines and chemokines and invasion of immune cells and contributes to the development of various chronic diseases [1]. The activation of PRRs is closely associated with the onset and progression of obesity-related metabolic diseases by responding to metabolic stress. In particular, activation of Toll-like receptor (TLR)-2 and TLR-4 is associated with obesity-mediated insulin resistance and the pathogenesis of metabolic diseases. Saturated fatty acids trigger insulin resistance by activating TLR-2 and TLR-4 [2,3]. In contrast, omega-3 polyunsaturated fatty acids, such as docosahexaenoic acid and eicosapentaenoic acid, prevent the activation of TLRs by lipopolysaccharides or saturated fatty acids. The NOD-, LRR-, and pyrin domain-containing protein 3 (NLRP3) inflammasome increases the secretion of inflammatory cytokines through the activation of caspase-1. NLRP3 plays an important role in the progression of metabolic disorders, including obesity-induced insulin resistance and the development and severity of gout, nonalcoholic fatty liver disease, and type 2 diabetes [4]. Thus, it has been proposed that TLR inhibitors and NLRP3 inhibitors exert potential therapeutic effects on metabolic diseases such as gout and NAFLD [5,6].

RIG-I-like receptors are cytosolic PRRs found in all cell types, including immune cells, and they are vital for the immune recognition of and response to most RNA viruses [7]. The RLR family includes retinoic acid-inducible gene-I (known as RIG-I or DDX58), melanoma differentiation-associated gene 5 (MDA5), and laboratory of genetics and physiology 2 (LGP2). RIG-I and MDA5 contain a DExD/H box RNA helicase domain, a C-terminal repressor domain (RD or CTD), and two caspase-recruiting domains (CARDs) necessary for signaling. In contrast, LGP2 contains no CARDs and functions as the dominant negative regulator of RIG-I and DMA5 [8,9,10]. In addition to the role of RIG-I in antiviral immunity, the possible role of RIG-I in the regulation of metabolic stress has been suggested. The level of RIG-I was elevated by glucolipotoxicity in a pancreatic β cell line, and RIG-I inhibited the proliferation of pancreatic β cells through G1 cell cycle arrest [11]. Zhang et al. reported that lactate, a product of glucose metabolism, negatively regulated RIG-I signal activation, resulting in the downregulation of interferon production. This indicates that RIG-I activation can be regulated by metabolic stress [12]. However, the role of the RLR family in the regulation of obesity has not been elucidated. Therefore, we investigated whether RIG-I or MDA5 was involved in the regulation of obesity-induced metabolic stress using high-fat diet (HFD)-fed knockout mice.

## 2. Results

### 2.1. RIG-I Deficiency Enhances High-Fat Diet-Induced Obesity in Mice

**Figure 1:**
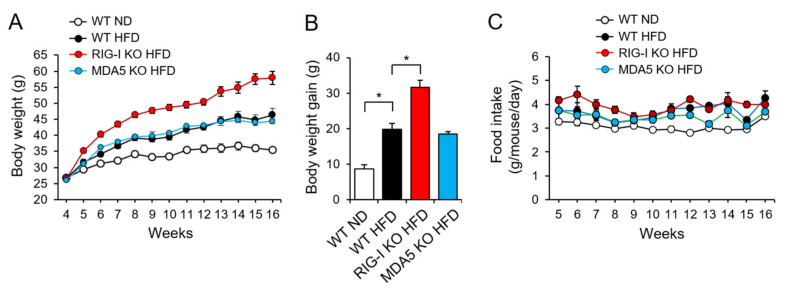
RIG-I deficiency exacerbates high-fat diet-induced obesity in mice. Wild-type (WT), RIG-I knockout (KO), or MDA5 KO mice were fed a normal chow diet (ND) or a high-fat diet (HFD) for 12 weeks. (**A**) Total body weight. (**B**) Body weight gain at 12-weeks. (**C**) Average food intake. The values represent the means ± SEM (*n* = 4–5). * *p* < 0.05.

We investigated whether RIG-I played a role in high-fat diet-induced body weight gain in mice. Wild-type (WT), RIG-I knockout (KO), or MDA5 KO mice were fed an HFD for 12 weeks. Mice fed an HFD showed higher body weight than mice fed a normal diet (ND) during the diet period (Figure 1A). Notably, RIG-I KO mice fed HFD (RIG-I KO HFD) showed a greater increase in body weight gain than WT mice fed HFD (WT HFD) at 12 weeks, while the body weight gain in MDA5 KO mice fed HFD (MDA5 KO HFD) was similar to that in WT HFD mice (Figure 1B). Food consumptions in the HFD groups were generally greater than that in the ND group (Figure 1C). In addition, the RIG-I KO HFD group showed slightly higher food consumption than the other HFD groups initially. However, the food consumption over the 12-week period was not significantly different among the three HFD groups (Figure 1C). The results show that RIG-I deficiency promotes high-fat diet-induced obesity in mice.

**Figure 2:**
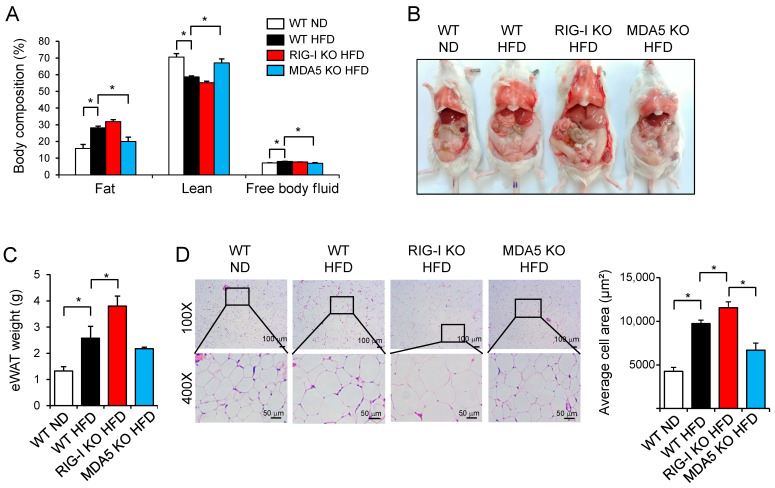
RIG-I deficiency increases fat deposition in mice fed a high-fat diet. (**A**) Body composition was analyzed in mice described in Figure 1. (**B**) Representative pictures of mice at the end of the 12-week experiment. (**C**) Epididymal white adipose tissue (eWAT) weight. (**D**) eWATs were stained with hematoxylin and eosin (H&E) (100×, 400×). Bar graphs represent the average cell size area of each group. The values in bar graphs represent the means ± SEM (*n* = 4–5). * *p* < 0.05.

Body composition analysis showed that the fat composition in WT HFD mice was greater than that in WT ND mice, and fat mass was slightly increased in RIG-I KO HFD mice compared to WT HFD mice (Figure 2A). Lean body mass was lower in the WT HFD mice than in the WT mice fed an ND (WT ND) (Figure 2A). In addition, RIG-I KO HFD mice exhibited a marginal decrease in lean body mass compared to WT HFD mice (Figure 2A). In contrast, the fat and lean body compositions of MDA5 KO HFD mice were similar to those of WT ND mice (Figure 2A). Consistently, RIG-I KO HFD mice exhibited a hypertrophic phenotype (Figure 2B). The epididymal white adipose tissue (eWAT) weight was higher in RIG-I KO HFD mice than in WT HFD mice (Figure 2C). Histological analysis showed that the eWAT of RIG-I KO HFD mice had greater adipocytes than that of WT HFD mice (Figure 2D). The liver weight increase in RIG-I KO HFD mice was higher than that in WT ND mice although the liver weight increase in RIG-I KO HFD mice was not significantly greater than that in WT HFD mice (Figure S1, Supplementary Materials). Hepatic triglyceride (TG) levels were increased in RIG-I KO HFD mice compared with WT ND mice (Figure S2, Supplementary Materials).

### 2.2. RIG-I Deficiency Aggravates High-Fat Diet-Induced Insulin Resistance

**Figure 3:**
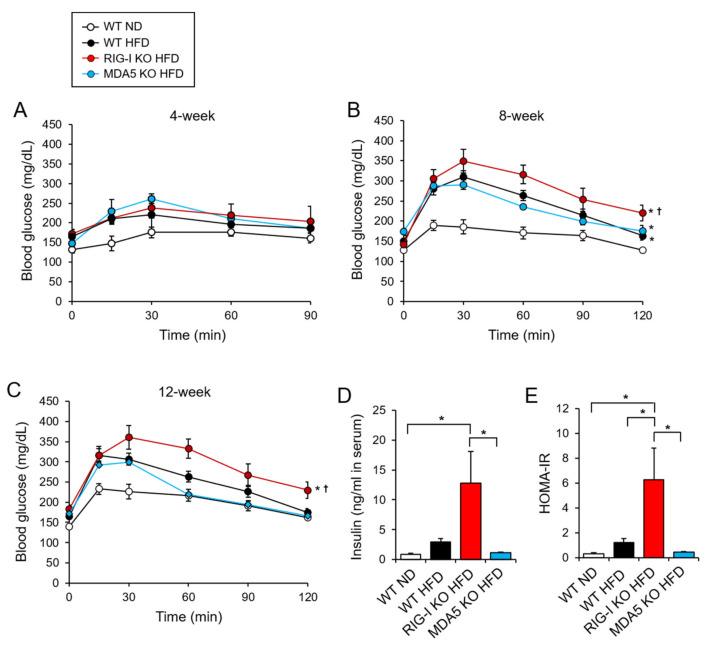
RIG-I deficiency aggravates high-fat diet-induced insulin resistance. (**A**–**C**) Intraperitoneal glucose tolerance tests were performed. Glucose solutions (2 g/kg) were administered via intraperitoneal injection after a 4-h fast. Blood glucose levels were measured at 0, 15, 30, 60, 90, and 120 min after the glucose challenge at 4, 8, and 12 weeks on diet. (**D**,**E**) The levels of fasting serum insulin and serum homeostasis model assessment of insulin resistance (HOMA-IR) at 12 weeks. The values represent the means ± SEM (*n* = 5). (**A**–**C**) * *p* < 0.05 compared with WT ND mice. ^†^ *p* < 0.05 compared with WT HFD mice. (**D**,**E**) * *p* < 0.05.

Next, we investigated whether RIG-I deficiency affected metabolic syndrome. Glucose tolerance tests were performed at 4, 8, and 12 weeks after a high-fat diet. At 4 weeks, the three HFD groups showed slightly impaired glucose tolerance compared to the WT ND group (Figure 3A, Table 1). Glucose tolerance was markedly impaired in the three HFD groups at 8 and 12 weeks (Figure 3B,C, Table 1). Among the three HFD groups, the glucose tolerance of the RIG-I KO HFD mice was impaired the most (Figure 3B,C, Table 1). In contrast, the glucose intolerance of MDA5 KO HFD mice was not greater than that of WT HFD mice (Figure 3B,C, Table 1). At 12 weeks, RIG-I KO HFD mice had significantly higher fasting serum insulin levels than WT HFD and MDA5 KO HFD mice (Figure 3D). Homeostatic Model Assessment for Insulin Resistance (HOMA-IR) was also significantly higher in RIG-I KO HFD mice than in other HFD mice (Figure 3E). These results show that RIG-I deficiency aggravates glucose intolerance induced by a high-fat diet.

### 2.3. RIG-I Is Required for IFN-β Production in Response to ER Stress

**Figure 4:**
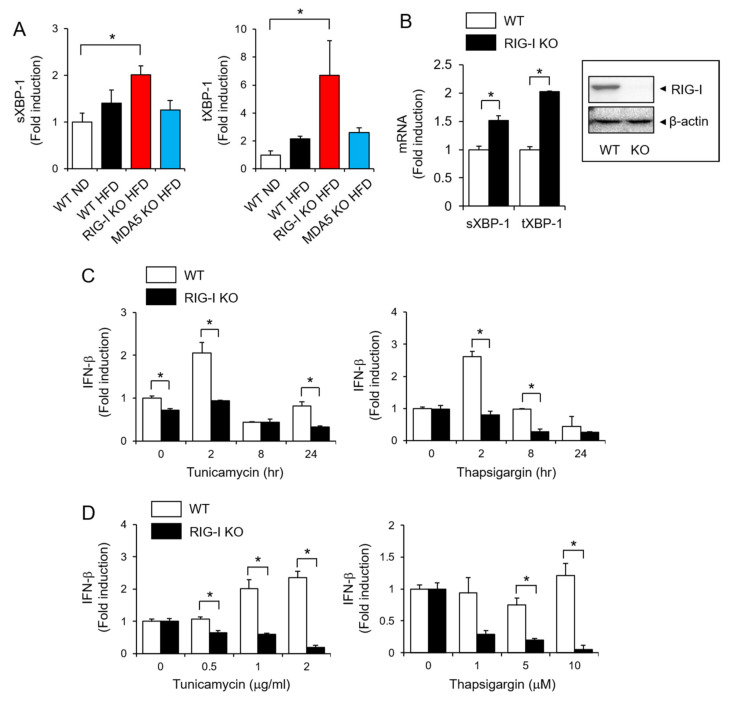
RIG-I is required for IFN-β production in response to ER stress. (**A**) Liver homogenates were prepared from mice after 12 weeks of treatment as described in Figure 1. The mRNA levels of sXBP-1 and tXBP-1 were determined by qRT–PCR. (**B**) Primary mouse hepatocytes isolated from WT or RIG-I KO mice were treated with tunicamycin (1 μg/mL) for 16 h. The mRNA levels of sXBP-1 and tXBP-1 were determined by qRT–PCR. The protein expression of RIG-I was determined by immunoblotting of the hepatocytes. The original immunoblotting is provided in Figure S3, Supplementary Materials. (**C**) Primary mouse hepatocytes isolated from WT or RIG-I KO mice were treated with tunicamycin (2 μg/mL) or thapsigargin (10 μM) for the indicated times. The mRNA levels of IFN-β were determined by qRT–PCR. (**D**) Bone marrow-derived macrophages from WT or RIG-I KO mice were treated with tunicamycin or thapsigargin for 24 h. The mRNA levels of IFN-β were determined by qRT–PCR. The values represent the means ± SEM (*n* = 3–5). * *p* < 0.05.

Endoplasmic reticulum (ER) stress is closely associated with the development of obesity [13]. Therefore, we investigated the relationship between RIG-I and ER stress in high-fat diet-induced metabolic stress. The expression of sXBP-1 and tXBP-1 was significantly en-hanced in livers isolated from RIG-I KO HFD mice compared with livers from WT ND mice (Figure 4A). Similarly, treatment of hepatocytes with tunicamycin, an ER stress in-ducer, showed that the expression of sXBP-1 and tXBP-1 was enhanced in RIG-I-deficient hepatocytes compared with wild-type hepatocytes (Figure 4B). These results suggest that RIG-I deficiency potentiates ER stress in cells.

ER stress is accompanied by a viral infection-induced interferon (IFN) response, and ER stress induces type I IFN expression [14]. Treatment with the ER stress inducers tuni-camycin and thapsigargin increased the expression of IFN-β in mouse primary hepato-cytes, but this induction was abolished in RIG-I-deficient hepatocytes (Figure 4C). The role of RIG-I is well known for inducing anti-viral immunity. Therefore, we investigated whether ER stress-induced IFN-β production was affected in RIG-I-deficient immune cells. Consistently, the induction by tunicamycin or thapsigargin was abolished in RIG-I-deficient primary mouse macrophages (Figure 4D). These results show that RIG-I is required for ER stress-induced IFN-β expression.

## 3. Discussion

Our results show that RIG-I deficiency promotes obesity and insulin resistance induced by a high-fat diet. To the best of our knowledge, this is the first report showing the regulatory role of RIG-I in metabolic stress, obesity, and insulin resistance. Obesity largely results from a chronic imbalance between energy intake and expenditure, and it is a strong risk factor for a number of metabolic diseases, including type 2 diabetes mellitus (T2D), hyperlipidemia, atherosclerosis, and nonalcoholic fatty liver disease (NAFLD).

PRRs induce inflammation, thereby contributing to the initiation and progression of obesity and related metabolic stress. For example, activation of Toll-like receptors (TLRs) or NLRP3 inflammasome is known to instigate the development of obesity and type 2 diabetes. TLR4 knockout mice were protected from high-fat diet-induced insulin resistance [3]. Reduction of NLRP3 expression in adipose tissue was correlated with improvement of insulin sensitivity in type 2 diabetes patients [15]. In contrast, our results show that RIG-I receptor deficiency promotes obesity. There are reports describing the protective role of type I IFNs against metabolic stress. We speculate that reduced type I IFNs production might mediate the enhancement of obesity and insulin resistance in RIG-I-deficient animals. RIG-I activation culminates in the production of type I interferons (IFN), IFN-α, and IFN-β, which are important regulators of both innate and adaptive immune responses. Type I interferons contribute to the pathology of obesity and related metabolic disorders, possibly mediated by regulating adipocyte inflammatory potential. A study using interferon receptor 1 (Ifnar1) knockout mice showed that type I IFN signaling in adipocytes and hepatocytes protected against high-fat diet-induced metabolic dysregulation or methionine-choline-deficient (MCD) diet-induced hepatic disease [16]. Overexpression of the Ifnb gene inhibited inflammation in adipose tissue and reduced adipose tissue expansion and body weight gain in mice fed a high-fat diet [17]. These results suggest a protective role of type I IFNs against obesity-related metabolic disorders. In contrast, other studies have reported a role of type I IFNs in promoting metabolic stress. Chan et al. reported that type I IFNs amplified adipocyte inflammatory signatures and were implicated in obesity-mediated metabolic disorders in humans [18]. Ghazarian et al. showed that type I IFNs promoted the accumulation of pathogenic CD8+ T cells in the liver, leading to glucose dysregulation in diet-induced obesity in mice [19]. Our results show that RIG-I deficiency reduces ER stress-induced IFN-β expression in hepatocytes and macrophages, which is caused by the aggravation of glucose dysregulation induced by a high-fat diet in mice. In addition, the levels of IFN-α and IFN-β were lower in livers from RIG-I KO HFD animals than in livers from WT HFD animals (Figure S4, Supplementary Materials). These results suggest that IFN-β plays a protective role in obesity-induced metabolic dysregulation in the RIG-I-mediated signaling pathway.

Endoplasmic reticulum (ER) stress is considered a cause of metabolic diseases. In animal models with obesity and type 2 diabetes, increased endoplasmic reticulum stress was observed in tissues related to metabolic regulation, such as the hypothalamus, liver, and adipose tissue [20,21,22]. Excessive fatty acids and glucose impair cellular metabolism and activate cellular stress signals, including ER stress, mitochondrial dysfunction, and oxidative stress, in multiple organs. Glucotoxicity has an indirect effect on the ER through protein succination by fumarate produced via mitochondria under excessive glucose levels [23]. In particular, high fructose levels in the liver and pancreas can lead to insulin resistance and diabetes due to ER stress [24]. XBP1 is one of the key regulators of ER stress-mediated cellular responses and regulates a subset of genes encoding ER-resident proteins of the unfolded protein response (UPR) [25,26]. XBP1 is also important for homeostasis and is especially essential for lipid synthesis in the liver [27]. ER stress culminates in unfolded protein responses (UPR), accompanied by three regulatory signaling sensors, protein kinase RNA-like ER kinase (PERK), inositol-requiring protein 1α (IRE1α), and activating transcription factor 6 (ATF6). Activated IRE1 induces cleavage of XBP1u into the active splice form, XBP1s. In addition, IRE1 induces the phosphorylation of Jun NH_2_-termianl kinase (JNK) [28], suggesting that the important role of the IRE1-XBP1 pathway in the regulation of insulin signaling and glucose metabolism. Our results show that the expression of XBP-1 was elevated in RIG-I-deficient mouse livers, suggesting that ER stress is augmented by RIG-I deficiency. XBP1 has been implicated in the synergistic production of type 1 interferons (IFNs) in response to a combination of ER stress and Toll-like receptor (TLR) signaling in macrophages [29,30]. However, despite the increase in XBP1 levels in the RIG-I KO HFD group, IFN-β levels were downregulated by RIG-I KO, suggesting that in RIG-I signaling pathways, XBP-1 is not required for IFN-β expression.

The role of RIG-I in the regulation of antiviral immunity is well known. RIG-I activation is involved in the development of autoimmune diseases such as systemic lupus erythematosus (SLE) [31] and atypical Singleton-Merten Syndrome (SMS) [32,33]. Our study presents a novel role of RIG-I in the development of obesity and metabolic disorders. We observed that the expression of RIG-I protein was decreased in livers isolated from mice fed with high-fat diet compared with livers from mice fed with a normal diet (Figure S5, Supplementary Materials). In addition, Frietze et al. showed that RIG-I protein levels were decreased in livers isolated from the NAFLD/NASH mouse model induced by a choline-deficient, L-amino acid-defined high-fat diet (CDAHFD) [34]. Palmitic acid, a saturated fatty acid, reduced RIG-I activity, showing that RIG-I activity is regulated by lipotoxicity [34]. Activation of RIG-I induced an autophagic response to protect against lipotoxicity, suggesting that regulation of RIG-I activity could be a strategy to treat NAFLD [34]. These suggest that searching the modulators of RIG-I activity would provide a therapeutic strategy for the treatment of metabolic diseases.

## 4. Materials and Methods

### 4.1. Animals

RIG-I/MDA5 double knockout (KO) mice were provided by Shizuo Akira (Osaka University) and were bred to produce heterozygous and homozygous single-KO (RIG-I KO or MDA5 KO) mice. All animals received humane care according to the criteria of the “Guide for the Care and Use of Laboratory Animals” prepared by the National Academy of Sciences and published by the National Institutes of Health (NIH publication 86-23 revised 1985).

### 4.2. Cell Culture

Primary hepatocytes were isolated from mice using collagenase perfusion, as previously described [35]. Hepatocytes were cultured in Dulbecco’s Modified Eagle’s Medium (DMEM) supplemented with 25 mM glucose, 10% (*v*/*v*) fetal bovine serum (FBS, Invitrogen, Carlsbad, CA, USA), 10,000 units/mL penicillin, and 10,000 µg/mL streptomycin (Invitrogen). Based on trypan blue staining, the survival rate was >90% for all preparations.

Bone marrow-derived primary macrophages were prepared after bone marrow isolation from ICR mice as previously described [6]. Macrophages were cultured in DMEM supplemented with 10% (*v*/*v*) FBS, 10,000 units/L penicillin, and 10,000 µg/mL streptomycin (Invitrogen).

### 4.3. Reagents

Tunicamycin (T7765) and thapsigargin (T9033) were purchased from Sigma-Aldrich (St. Louis, MO, USA). The concentrations of tunicamycin and thapsigargin for cell treatment were determined according to the previous reports [36,37,38].

### 4.4. A High Fat Diet-Induced Obesity Mouse Model

Wild-type, RIG-I KO, or MDA5 KO ICR male mice received a normal chow diet or high-fat diet obtained from Research Diets (New Brunswick, NJ, USA) for 12 weeks, starting at the age of 4 weeks. The normal chow diet is composed of 20% protein, 70% carbohydrates, and 10% fat by kcal. The high-fat diet is composed of 20% protein, 35% carbohydrates, and 45% fat by kcal. All experiments were carried out using wild-type male littermates as controls.

### 4.5. Analysis of Whole-Body Composition

Whole-body composition (fat, lean tissue, and free body fluid) was assessed in live mice by LF50 from Bruker (Billerica, MA, USA). Technical support for this procedure was provided by the Animal Care Committee at Seoul University College of Veterinary Medicine, Korea Mouse Phenotyping Center (KMPC).

### 4.6. Histological Analysis

Epididymal white adipose tissues (eWATs) were fixed in 10% buffered formalin and embedded in paraffin. Using a Finesse ME Microtome (Thermo, Waltham, MA, USA), the samples were cut at 3 μm thickness and stained with hematoxylin and eosin for histological examination.

### 4.7. Intraperitoneal Glucose Tolerance Test (IPGTT)

Mice were fasted for 4 h, and fasting blood glucose was measured. Then, glucose solutions (2 g/kg) were administered via intraperitoneal injection. The blood glucose levels were measured at 15, 30, 60, and 120 min after the glucose challenge as previously described [39]. The blood insulin levels were assayed using rat/mouse insulin ELISA kits (Merck, Darmstadt, Germany). The concentration range for the insulin standard curves was 0.2 to 10 ng/mL.

### 4.8. Quantitative Real-Time Polymerase Chain Reaction (qPCR) Analysis

This analysis was performed as previously described [40]. Total RNA was extracted using TRIzol reagent (Invitrogen) according to the manufacturer’s instructions. One microgram of total mRNA was reverse transcribed to prepare complementary DNA (cDNA) using an ImProm-II™ Reverse Transcription System (Promega Corporation, Madison, WI, USA). The following primers were used: Ifn-β(NM_010510), 5′-TCC-AAG-AAA-GGA-CGA-ACA-TTC-G-3′ and 5′-TGA-GGA-CAT-CTC-CCA-CGT-CAA-3′; sXBP-1(NM_001271730.1), 5′-GAG-TCC-GCA-GCA-GGT-G-3′ and 5′-GTG-TCA-GAG-TCC-ATG-GGA-3′; tXBP-1(NM_013842.3), 5′-AAG-AAC-ACG-CTT-GGG-AAT-GG-3′ and 5′-ACT-CCC-CTT-GGC-CTC-CAC-3′; β-actin, 5′-TCA-TGA-AGT-GTG-ACG-TTG-ACA-TCC-GT-3′ and 5′-TTG-CGG-TGC-ACG-ATG-GAG-GGG-CCG-GA-3′. The specificity of the amplified PCR products was assessed using melting curve analysis, and the mRNA expression levels of each gene were normalized to the corresponding β-actin levels.

### 4.9. Immunoblotting Analysis

Immunoblotting was performed as previously described [41]. Primary mouse hepatocytes were lysed in RIPA buffer containing 50 mM Tris-HCl (pH 7.5), 150 mM NaCl, 0.5% Nonidet P-40, and 0.1% SDS supplemented with protease inhibitors (10 µg/mL leupeptin, 10 µg/mL pepstatin A, 10 µg/mL aprotinin, and 1 mM 4-(2-aminoethyl) benzenesulfonyl fluoride) and phosphatase inhibitors (1 mM NaF and 1 mM Na_3_VO_4_). Proteins were applied to SDS-PAGE and transferred to PVDF membranes. After blocking with 5% skim milk (Difco^TM^, Becton, Dickinson and Company, Franklin Lakes, NJ, USA), the membranes were incubated with an antibody for RIG-I or β-actin followed by incubation with a peroxidase-conjugated secondary antibody. Blots were visualized by the ECL system (BioNote Inc., Gyeonggi-do, Korea).

### 4.10. Statistical Analysis

All data are presented as the mean ± standard error mean (SEM) of three or more independent experiments. Statistical evaluation was conducted using one-way analysis of variance followed by Duncan’s multiple range test to determine any significant differences. A *p* value < 0.05 was considered significant.

## 5. Conclusions

In conclusion, we present a novel regulatory role of RIG-I in the development of obesity and metabolic disorders, further suggesting that the modulation of RIG-I activity would provide an effective therapeutic strategy for the treatment of metabolic diseases.

## Figures and Tables

**Table 1:** Area under the curve (AUC) of intraperitoneal glucose tolerance tests.

	4-Week			8-Week			12-Week		
WT ND	646.5	±	42.79	838.3	±	46.96	1018	±	60.07
WT HFD	868.7	±	54.32 *	1227	±	48.95 *	1282	±	71.26 *
RIG KO HFD	856.4	±	80.41 *	1406	±	86.43 *^,†^	1483	±	101.4 *^,†^
MDA KO HFD	802.7	±	73.32 *	1188	±	33.24 *	1175	±	59.71 *

* Different from WT ND, *p* < 0.05. ^†^ Different from WT HFD, *p* < 0.05.

## Data Availability

Data is contained within the article and Supplementary Materials.

## Supplementary Materials

The following are available online at https://www.mdpi.com/article/10.3390/ph14111178/s1, Figure S1: Liver weights of wild-type (WT), RIG-I knockout (KO), or MDA5 KO mice fed normal diet (ND) or high fat diet (HFD); Figure S2: Hepatic triglyceride (TG) levels of wild-type (WT), RIG-I knockout (KO), or MDA5 KO mice fed normal diet (ND) or high fat diet (HFD); Figure S3: RIG-I expression in primary hepatocytes isolated from wild-type (WT) or RIG-I-knockout (KO) mice; Figure S4: Hepatic IFN-α and IFN-β levels of wild-type (WT), RIG-I knockout (KO), or MDA5 KO mice fed normal diet (ND) or high fat diet (HFD); Figure S5: RIG-I expression in livers isolated from of wild-type mice fed normal diet (ND) or high fat diet (HFD).
