# Supplementary material for: RIG-I Deficiency Promotes Obesity-Induced Insulin Resistance
Source: Pharmaceuticals (Basel). 2021 Nov 17;14(11):1178. doi: 10.3390/ph14111178 (PMC8624253; doi:10.3390/ph14111178)
Supplement: Supplementary file 1 [file pharmaceuticals-14-01178-s001.zip › pharmaceuticals-1398728-supplementary.pdf]

Supplementary Materials

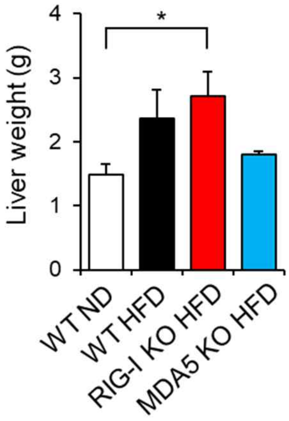

Figure S1. Liver weights of wild-type (WT), RIG-I knockout (KO), or MDA5 KO mice fed normal diet (ND) or high fat diet (HFD). Liver weights were measured after 12 weeks of treatment as described in Figure 1. The values in bar graphs represent means ± SEM (n=4–5). *$p < 0.05$.

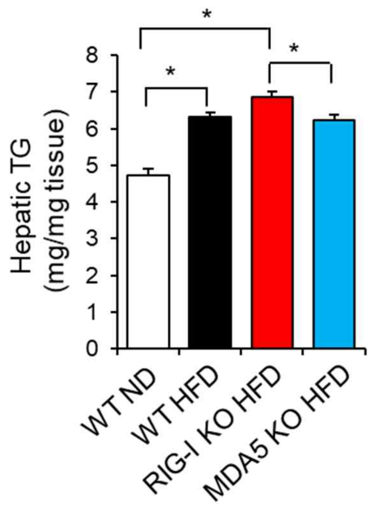

Figure S2. Hepatic triglyceride (TG) levels of wild-type (WT), RIG-I knockout (KO), or MDA5 KO mice fed normal diet (ND) or high fat diet (HFD). Liver samples were obtained after 12 weeks of treatment as described in Figure 1. The values in bar graphs represent means ± SEM (n=4–5). *$p < 0.05$.

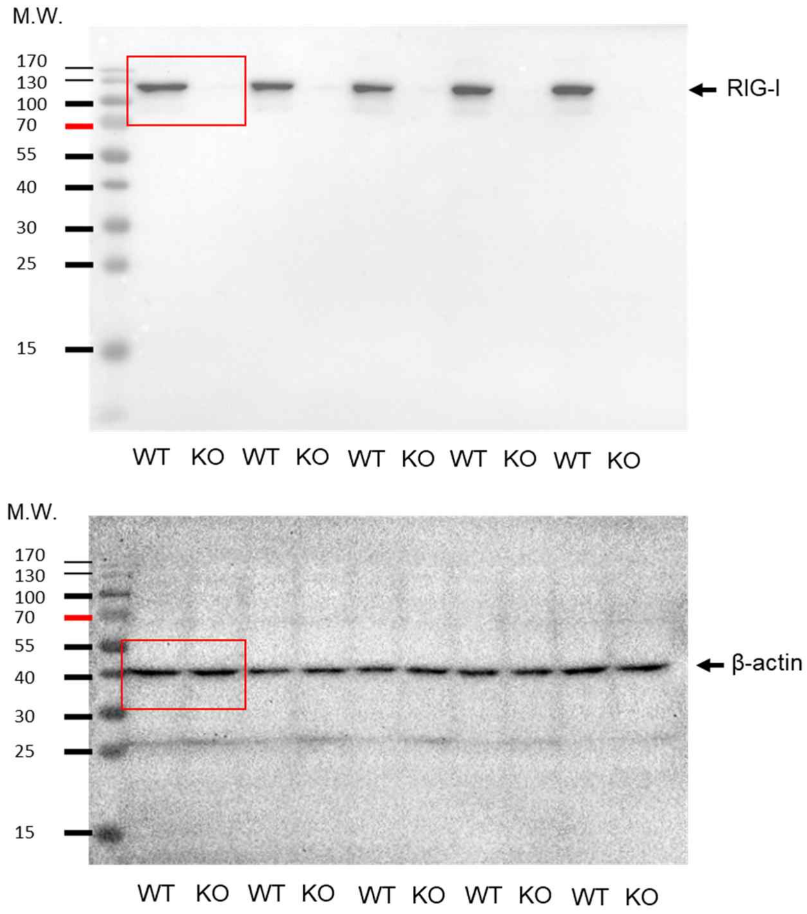

Figure S3. RIG-I expression in primary hepatocytes isolated from wild-type (WT) or RIG-I-knockout (KO) mice. Protein expression of RIG-I and β-actin was determined by immunoblotting. The bands in red boxes are presented in Figure 4B.

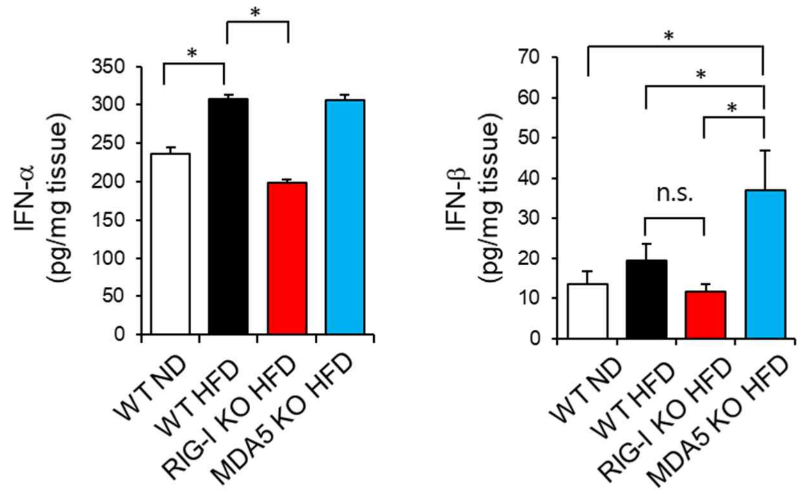

Figure S4. Hepatic IFN-α and IFN-β levels of wild-type (WT), RIG-I knockout (KO), or MDA5 KO mice fed normal diet (ND) or high fat diet (HFD). Liver samples were obtained after 12 weeks of treatment as described in Figure 1. The levels of IFN-α and IFN-β were determined by ELISA. The values in bar graphs represent means ± SEM (n=4–5). *$p < 0.05$.

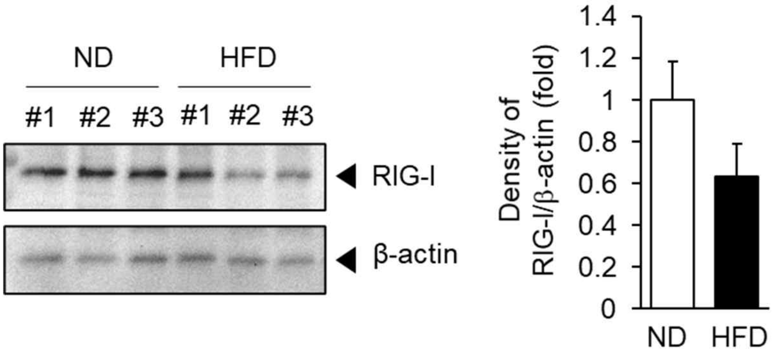

Figure S5. RIG-I expression in livers isolated from of wild-type mice fed normal diet (ND) or high fat diet (HFD). Protein expression of RIG-I and β-actin was determined by immunobloting. The numbers represent individual animals. The graph represents density of RIG-I/β-actin. The values in bar graphs represent means ± SEM (n=3).
